# Large aortic aneurysm mimicking a cardiac tumor

**DOI:** 10.1186/1476-7120-8-33

**Published:** 2010-08-17

**Authors:** Zhen-Yu Liao, Jui-Peng Tsai, Jen-Yuan Kuo, Chung-Lieh Hung

**Affiliations:** 1 Division of Cardiology, Department of Internal Medicine, Mackay Memorial Hospital, Taipei, Taiwan, No 92, Chung-Shan North Road, 2nd section, Taipei, Taiwan, ROC; 2 Department of Medicine, Mackay Medical College, and Mackay Medicine Nursing and Management College, Taipei, Taiwan, No 92, Chung-Shan North Road, 2nd section, Taipei, Taiwan, ROC; 3 Cardiovascular Division, Brigham and Women's Hospital, Boston, MA, No 92, Chung-Shan North Road, 2nd section, Taipei, Taiwan, ROC

## Abstract

**Introduction:**

Extrinsic left atrial compression caused by a displaced, crooked descending thoracic aorta is rare. This anomaly may mimic primary cardiac tumors or metastatic neoplasms from the first look.

**Case presentation:**

We reported a 78-year-old woman presented to our emergency room with back pain, increased exercise intolerance and intermittent angina. She also had one syncopal event 1 month ago and gastric cancer post gastrectomy history. Subsequent chest plain film showed no mediastinum widening.

Two-dimensional echocardiography was performed and revealed a heterogeneous mass as large as 2.3 × 2.4 cm occupying the left atrium (LA). Three-dimensional echocardiography vividly demonstrated that LA was constrained between the aortic valve (AV) and a luminal structure with pulsatile character suggestive of the aorta.

**Conclusions:**

We successfully demonstrated the detailed structure and location of an anomalous descending aorta on the oblique imaging plane of RT-3DE, which may not be readily available by traditional 2D method.

## Introduction

Primary tumors of the heart are rare, and its clinical diagnosis can be difficult. While the classic signs and symptoms of left atrial myxoma are noteworthy, most of these tumors present with non-specific symptoms - either of a constitutional nature, or related to right- or left-sided congestion [1].

Simulating lesions including metastatic extension of mediastinum tumor and bronchogenic cyst should also be suspected. Anterior mediastinum masses tend to compress the right heart chambers, while posterior masses impinge on or compress the left atrium or ventricle, particularly the former [2].

Extrinsic left atrial compression caused by a displaced, crooked descending thoracic aorta is rare. This anomaly may mimic primary cardiac tumors or metastatic neoplasms from the first look.

## Case presentation

On January 29th 2010, a 78-year-old woman presented to our emergency room with back pain, increased exercise intolerance and intermittent angina. She also had one syncopal event 1 month ago. Her past medical history included gastric cancer post gastrectomy.

**Figure 1:**
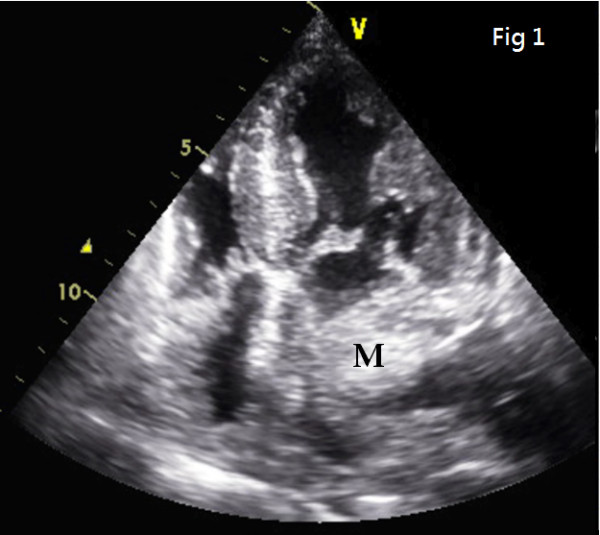
**Two-dimensional echocardiography view: a heterogeneous mass as large as 2.3 × 2.4 cm occupying the left atrium (LA) is revealed**.

Upon physical examination, blood pressure was 123/75 mmHg and heart rate was 70 beats/min. Surface electrocardiogram showed normal sinus rhythm with left anterior hemiblock. Initial laboratory data such as biochemistry, electrolytes and blood cell count were normal. Subsequent chest plain film showed no mediastinum widening. Two-dimensional echocardiography was performed at bedside and revealed a heterogeneous mass as large as 2.3 × 2.4 cm occupying the left atrium (LA) (Fig. 1).

**Figure 2:**
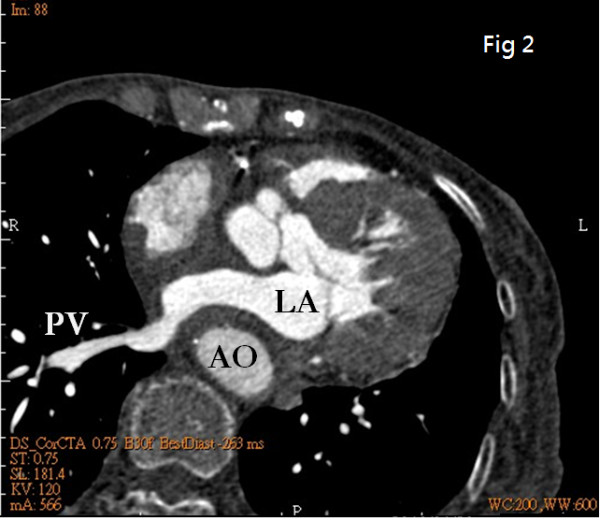
**CT images: a displaced descending aorta (AO) constricting the left atrium (LA) is revealed**.

**Figure 3:**
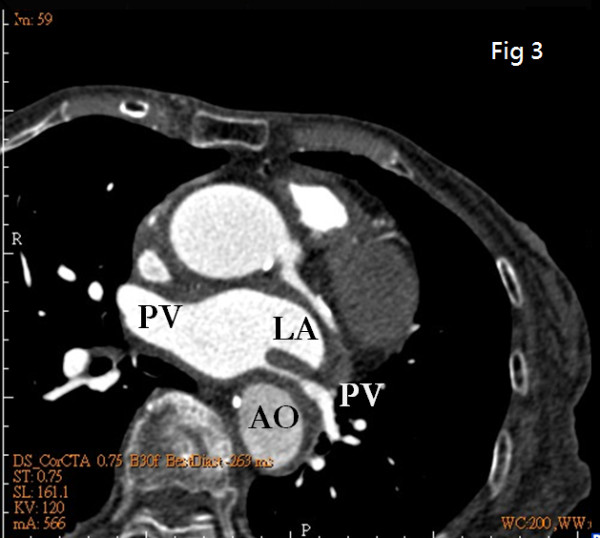
**Another plane of CT images: the left pulmonary vein critically squeezed is clearly demonstrated**.

**Figure 4:**
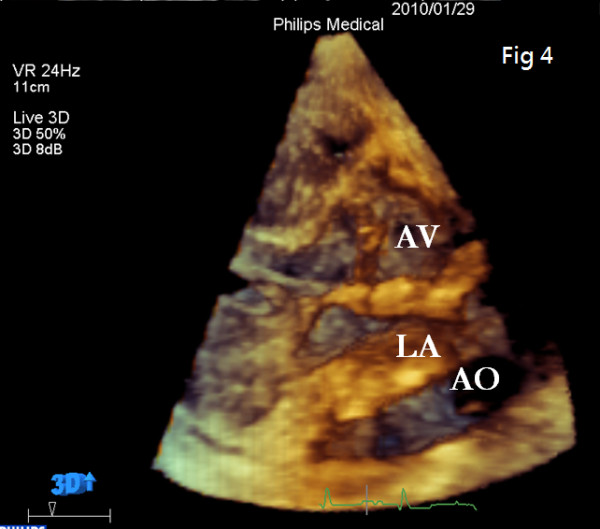
**Three-dimensional echocardiography view: LA is constrained between the aortic valve (AV) and a luminal structure with pulsatile character is suggestive of the aorta**.

To further determine the nature of this LA mass, computed tomography (CT) was done. Interestingly, neither primary cardiac tumor nor metastatic tumor was shown, and there was no lymphadenopathy. Instead, CT images revealed a displaced descending aorta (AO) constricting the left atrium (LA) (Fig. 2). Another plane clearly demonstrated that the left pulmonary vein was critically squeezed (Fig. 3). Three-dimensional echocardiography also vividly demonstrated that LA was constrained between the aortic valve (AV) and a luminal structure with pulsatile character suggestive of the aorta (Fig. 4) (Additional file 1).

After nearly 1 month of rehabilitation, she felt much better and was discharged. When seen in March 2010, the patient was symptom-free.

## Discussion

A distinction may be made between encroachment: distortion or partial displacement of one or more cardiac chambers by a contiguous mediastinal mass, without adverse hemodynamic effects, and compression: resulting in clinical manifestations such as tamponade. Nevertheless, the majority of patients are incidentally diagnosed by chest plain film or echocardiography. Two-dimensional echocardiography remains the routine standard for determining the nature of intra-cardiac and pericardial mass lesions, but its acoustic access to mediastinal structures is limited [3].

In previous literature, Morris et al [4] made an excellent systematic review on thoracic aortic aneurysms (TAA) complicated by LA compression or fistula formation affecting cardiac structures. Though recent pre-procedural imaging for pulmonary vein isolation of subjects with atrial fibrillation has reported variants of descending AO compressing on LA and PV, they have not been known to mimic tumor morphology [5,6]. Extensive imaging by CT or MRI is usually required for recognizing the precise spatial relationship between the PV, LA and AO, though the information obtained from 2D echocardiography can also be complementary.

## Conclusions

In cases of uncertain findings, real-time 3D echocardiography (RT-3DE) might be helpful in identifying the object in question by fully exploring adjacent cardiac structures at bed-side with relative ease and convenience. In our case, we successfully demonstrated the detailed structure and location of an anomalous descending aorta on the oblique imaging plane of RT-3DE, which may not be readily available by traditional 2D method.

## Consent

Written informed consent was obtained from the patient for publication of this case report and accompanying images. A copy of the written consent is available for review by Editor-in-Chief of this journal.

## Competing interests

The authors declare that they have no competing interests.

## Authors' contributions

ZYL carried out writing, literature search, and image editing. JPT and JYK made substantial contributions to analyze and interpret the patient data. CLH (corresponding author) participated in coordination writing, and image editing. All authors read and approved the final manuscript.

## Supplementary Material

Additional file 1 **Clips of three-dimensional echocardiography**. The data provided demonstration that LA was constrained between the aortic valve (AV) and a luminal structure with pulsatile character suggestive of the aorta. Click here for file
